# Curettage: Indications, Technique and Complications1Read before Cincinnati Chapter Alumni Associaton of Medical College of Ohio.

**Published:** 1907-04

**Authors:** John Miller

**Affiliations:** Cincinnati; Assistant to the Chair of Gynecology, Medical College of Ohio (University of Cincinnati)


### Curettage: Indications, Technique and Complications.[1]

BY JOHN MILLER, M. D., Cincinnati.
Assistant to the Chair of Gynecology, Medical College of Ohio (University of Cincinnati)

[*Lancet-Clinic, March 16, 1907.*]

OF the minor gynecological operations, none is done so often and with such varying technique as curettage of the uterus. It has become such a common practice that there are few physici-

---

1, Read before Cincinnati Chapter Alumni Associaton of Medical College of Ohio.

ans and general surgeons who will not attempt it. Ever since Recamier, in 1847, introduced the operation for the removal of hypertrophied mucous membrane of the uterus, various kinds of curettes and dilators have been invented. Like all surgical instruments, the advantage or disadvantage of the curette is largely dependent upon the man behind it, upon his judgment as to the wisdom of its employment in the individual case, and as to the manner of its employment.

The operation is indicated:

First, in cases of doubtful diagnosis, in order to differentiate a malignant from a benign tumor of the interior of the uterus.

Second, in chronic endometritis, whether manifested by excessive menstruation or hemorrhage between menstrual times, or by a profuse and continuous leucorrheal discharge, which has resisted local treatment.

Third, in uterine deviations which produce symptoms, and which deviations are to be corrected at the same time by suitable operations.

Fourth, in cases of chronic endometritis accompanied by inflammatory conditions of the tubes or ovaries, provided this pathology is also removed at the same time by some operative procedure.

Fifth, to remove retained products of conception.

Sixth, in inoperable cases of cancer to remove the disintegrating tissue.

In those cases of simple or catarrhal endometritis occurring in the virgin, whose uterus is of normal size or of the undeveloped type, which does not yield to constitutional treatment, as regular habits, diet, exercise and general tonics, I would consider dilatation and curettage in preference to any local treatment. The patient, in these cases, is to be anesthetised and a thorough dilatation and curettage, with tight packing, done. Local applications very often relieve the simple endometritis in those cases to which it is applicable.

The indications for curettage are many; the contra-indications, however, are few. I quote Reed: "The first contra-indication of curettage is non-experience in uterine surgery on the part of the operator. There is probably no manipulation in surgery for the proper practice of which more dexterity, more deftness, or more of that judgment which depends on the *tactus eruditus* is demanded than curettage."

I would consider as contra-indications for curettage, active pus in the tubes, ovaries or around the uterus; acute gonorrheal endometritis and acute septic endometritis, by which I mean infection following instrumentation, or operation about the cervix or body of the uterus. Those cases in which we have retained products of conception, with the general symptoms of chills, fever and rapid pulse, with a foul-smelling sanguino-purulent discharge, are considered as cases of sapremia, due to absorption of the putrefactive material. These cases are relieved by removing the offending particles of placenta or membrane by means of finger or curette.

With active pus in the tubes, ovaries or around the uterus, it is a dangerous procedure to curette the uterus, because the uterus is necessarily pulled down to the vaginal orifice, adhesions are broken up more or less, and pus set free. In all cases of curettage in which an abdominal section was made immediately after a curettement, we have always observed from one-half to one ounce of serum in the lower part of the pelvis, due probably to a weeping of the peritoneum as a result of the traction made upon the uterus. This serum would be an excellent culture medium for any germs set free during an operation.

In acute septic endometritis the germs have already entered the general circulation by the time we see the patient, and we might as well try to stop the effect of the vaccine virus or the systematic infection of syphilis by excising the site of the inoculation.

Some recent statistics of Whitridge Williams and Köenig show a reduction of mortality of from 22 per cent. after curettage to 5 per cent. by means of constitutional treatment. I have curetted these cases and have lost them very promptly with general septic infection, so that now I do not curette, but simply dilate the cervix thoroughly to facilitate drainage and wash out the cavity of the uterus with a warm antiseptic solution, and in some cases place a drainage-tube in the uterus. I wash out the uterus but once, and then bend all efforts to constitutional treatment. By means of the curette, whether you use the sharp, or much worse, if you use the dull, you open up the lymph-spaces and blood-vessels and absorption takes place so rapidly that nature is not able to take care of so great an amount of poison thrown into the system so suddenly that the patient is simply overwhelmed and the leukocytes are not equal to the task, and the patient dies of sepsis, whereas, if the lymph-spaces and blood-vessels are not opened up, the poison is introduced more gradually and nature is better able to fight it off. These cases, therefore, to my mind, are better treated constitutionally, and only that which is absolutely necessary done to the uterus. Gonorrheal infection also burrows deeply into the mucous and muscular tissue, and it is impossible to eradicate the disease in its acute stage by a curettage, and that which has been said of acute septic endometritis holds good in acute gonorrheal endometritis.

PREPARATION OF THE PATIENT.

The preparation of the patient should be carried out with as much care as though a major operation is to be performed, for one never knows in which case he is going to meet with an accident which might necessitate opening the abdomen. The patient's bowels should be well moved the day before, and the morning of the operation a large enema given. The vulva and vagina should be sterilised the night before and the hair around the vulva cut short. As a general anesthetic should be used whenever curetting the uterus, it is unnecessary to mention the fact that the stomach should be empty. After the patient is anesthetised and placed on the table in the lithotomy position, the vulva and vagina are again scrubbed with green soap and sterile water and flushed with bichloride solution and the bladder catheterised. The operator and assistants should have surgically clean hands and the instruments should be sterilised by boiling.

Before commencing the operation it is best to again examine the patient bimanually, to be sure of the position of the uterus and the conditions of the surrounding structures. Sterilised towels are placed over the thighs and a perineal apron is placed across the buttocks. The cervix is now exposed by means of Sims's specula, the anterior lips of the cervix grasped by a pair of volsellum forceps. The anterior speculum is now removed and the uterus drawn down toward the vulvar orifice and held steady, keeping the long axis of the volsellum in the long axis of the vagina, using them as tractors and not as levers. The cervical canal is now mopped out with bichloride solution. If the mucous plug which is in the cervix is very tenacious, dioxogen will often remove it easily. After the cervical canal is sterilised a uterine sound is passed into the cavity of the uterus to confirm the direction of its cavity, its size and shape. The cervical canal is then dilated by means of graduated sounds, or, as is our custom, first using the smaller-size Palmer dilator and following this by a Goodell-Ellinger dilator. Much harm can be done with this powerful instrument, and one must dilate slowly and cautiously, always using the pressure of the hand and not the thumbscrew. The cervical canal is to be dilated from a half to one and one-fourth inches. Many cases of perforation of the uterus occur at the time of the dilatation, quite often because the position of the uterus is not known, the curve of the dilator being in the wrong direction. The dilator makes a separation of the muscular fibres rather than a puncture. Now that the cervix is dilated, commence to curette, always using a fairly large size, sharp curette, with flexible shank, so that it can be bent to conform with the cavity of the uterus. Take some point as a starting place and make a clean stroke with the curette from the fundus to the internal os, being careful not to curette too deep at the internal os, for that is the place at which a stricture most frequently occurs. After you have gone over the entire cavity of the uterus with a large curette, use a small curette for the horns of the uterus. Then curette the cervical canal and around the external os. Mop the uterine cavity dry and free from mucous shreds with sterilised cotton, and then apply some antiseptic to the mucous membrane. Our preference is equal parts of tincture of iodine and carbolic acid. Before applying this to the inside of the uterus protect the vaginal portion of the cervix and the vagina by some cotton to catch any overflow of this solution. In most cases in which we do not open the abdomen, we pack the uterus tightly with iodoform gauze, which is left in the uterus from four to five days, removed sooner, however, if there is a rise of temperature to about 100 degrees. Before the patient is taken from the operating table the uterus is to be put in its normal position, a tampon being placed in the vagina in front of the cervix to retain it there. This tampon is removed the following day and a hot lysol douche given. The patient ought to remain in bed for a week.

### COMPLICATIONS.

Of the complications arising during curettage none is more frequent than perforation of the uterus by the dilator or curette. The instrument may pass into the peritoneal cavity or into the broad ligament. When recognised immediately and all aseptic precautions have been taken, there need be little fear of any serious complications. I would not mop out the uterus with any antiseptic, but would pack it with sterile gauze and place an ice-bag upon the uterus and keep the patient quiet. In all my cases in which the curette has passed into the uterus an unusual distance, I believe that I have perforated, and not that it was a temporary paralysis of the uterus, as recently suggested by Meier, of Philadelphia.

By means of the powerful metallic dilators the cervix might be torn; especially might this occur if the thumb-screw is used instead of dilating by means of the hand. The treatment in these cases suggests itself. Hemorrhage, especially in cases of malignancy, might give trouble, but can generally be controlled by means of gauze packing.

### CONCLUSIONS.

First, make an accurate pelvic diagnosis as to the size, position, mobility and consistency of the uterus, and determine the presence or absence of disease around the uterus.

Second, be surgically clean, use a general anesthetic and sharp curette.

Third, be competent to meet any accident which may arise.

